# Geodemographic analysis of socioeconomic area disparities in tuberculosis incidence in Osaka City, Japan

**DOI:** 10.1038/s41598-025-99711-4

**Published:** 2025-05-07

**Authors:** Kaori Yamamoto, Shouhei Takeuchi, Tomoki Nakaya, Naoya Fujiwara, Junji Seto, Jun Komukai, Yuko Tsuda, Hideki Yoshida, Takayuki Wada

**Affiliations:** 1 Division of Microbiology, Osaka Institute of Public Health, Address: 1-3-3, Nakamichi, Higashinari-ku, Osaka City, 537-0025 Osaka Japan; 2 Faculty of Nursing and Nutrition, University of Nagasaki, Nagayo, Nishisonogi, Japan; 3 Graduate School of Environmental Studies, Tohoku University, Sendai, Japan; 4 Department of Earth Science, Graduate School of Science, Tohoku University, Sendai, Japan; 5 Graduate School of Information Sciences, Tohoku University, Sendai, Japan; 6 PRESTO, Japan Science and Technology Agency, Kawaguchi, Japan; 7 Department of Microbiology, Yamagata Prefectural Institute of Public Health, Yamagata, Japan; 8 Department of Infectious Disease Control, Osaka City Public Health Office, Osaka, Japan; 9 Osaka City Health Bureau, Osaka, Japan; 10 Graduate School of Human Life and Ecology, Osaka Metropolitan University, Osaka, Japan; 11 Osaka International Research Center for Infectious Diseases, Osaka Metropolitan University, Osaka, Japan

**Keywords:** Geodemographics, Tuberculosis, Public health, Public health, Environmental sciences

## Abstract

Regional disparities in the incidence of tuberculosis (TB) pose several challenges to effective TB control. This study aimed to investigate such disparities in Osaka City, Japan, by analyzing the relationship between TB incidence and residents’ socioeconomic indicators. Using 42 indicators from the 2010 population census, the city was clustered into 15 social areas through a factor analysis, and TB incidence during 2012–2016 was compared across the areas in 4,852 cases. Two geographically adjacent areas (Area D and O) exhibited significantly higher TB rates, each with distinct social characteristics. Area D consisted of a high proportion of young, single individuals working in the service sector as well as foreigners. Area O included a high proportion of day laborers, secondary industry workers, and individuals with lower educational levels. TB occurred more frequently in foreign-born patients aged < 60 years, and it was detected during treatment for other diseases in Area D compared with other areas. However, a high proportion of retreated patients aged 40–79 years, with a shorter delay in TB detection, was observed in Area O. The variations in this study provide insights into the relationship between socioeconomic indicators and regional disparities in TB incidence in local settings.

## Introduction

Tuberculosis (TB), with 9.9 million cases and 1.5 million deaths reported worldwide in 2020^1^, continues to pose a major public health challenge. TB was the leading cause of death from a single infectious disease before the coronavirus disease (COVID-19) pandemic^2^. The implementation of non-pharmaceutical interventions in response to the COVID-19 crisis has contributed to a decrease in the number of TB cases; however, recent reports have indicated a resurgence of TB^1,3^. TB control is hindered by several unresolved challenges, including the potential ability of the causative agent (*Mycobacterium tuberculosis)* to reactivate after remaining latent following infection^4^. Effective treatment of TB warrants social care in the form of Direct Observation of Drugs and Therapy to ensure proper administration of anti-TB drugs and prevent the emergence and spread of drug-resistant strains^5^. The COVID-19 pandemic has strained public health resources, resulting in a resurgence in TB incidence and highlighting the continued critical need for effective TB control measures.

Metropolitan cities with large populations are particularly susceptible to the spread of TB owing to the presence of numerous places for gatherings, as well as the concentration of high-risk groups, such as socially vulnerable individuals, in certain areas^6^. Implementing a public health approach that considers the specific geographical characteristics of patients with TB is crucial for effective control. However, creating a public health system that adequately addresses the disparities within an urban city with a large population is a complex challenge^7^. Moreover, TB tends to be unevenly distributed in certain areas, in association with urban decline and corresponding inner-city challenges^8^. Ensuring equitable access to diagnosis and care tailored to local conditions is a key factor in addressing this challenge. Further research is warranted to understand the relationship between the social characteristics of local residents and TB incidence in small regional communities.

The World Health Organization has set a goal to achieve low endemicity of TB globally, with an incidence rate of < 10 per 100,000 people by 2035^9^. Japan successfully met this target in 2021^10^. Despite the steady decrease in TB incidence in Japan, Osaka City, the third largest municipality in Japan^11^, has exhibited a slower decrease in TB incidence compared to other areas^10^ (Supplementary Fig. 1), highlighting the need for further improvement in TB control measures. The implementation of measures aimed at reducing TB incidence in large cities, such as Osaka City, is crucial for advancing TB control efforts in the country.

Takatorige et al. (2000)^12^ analyzed TB incidence rates in 24 administrative wards of Osaka city and demonstrated disparities in the rate of decline among subgroups of wards defined based on the rate of unemployment, number of manufacturing industry workers, and number of construction industry workers. The study also revealed disparities in TB incidence rates within the city, highlighting the importance of adopting area-specific rather than uniform measures to control TB throughout the city. However, since this report, no further studies have been conducted to examine the regional distribution of TB in Osaka City.

Thus, we utilized small-area census data to divide Osaka city into socially segmented areas based on the geodemographic classification and examined the association between socioeconomic indicators of the areas and TB incidence. The study period encompassed the mid-endemic period, which is critical for clarifying the influence of regional characteristics and socioeconomic factors and for assessing the ongoing transition toward low endemicity. Regional segmentation based on geodemographic classification is a common method for analyzing the relationship between area-level socioeconomic factors and health outcomes^13^. This cross-sectional study will provide basic data for future TB control measures by identifying the characteristics of patients with TB and regional variations in urban areas.

## Results

### Attributes and geographic distribution of patients with TB

**Fig. 1:**
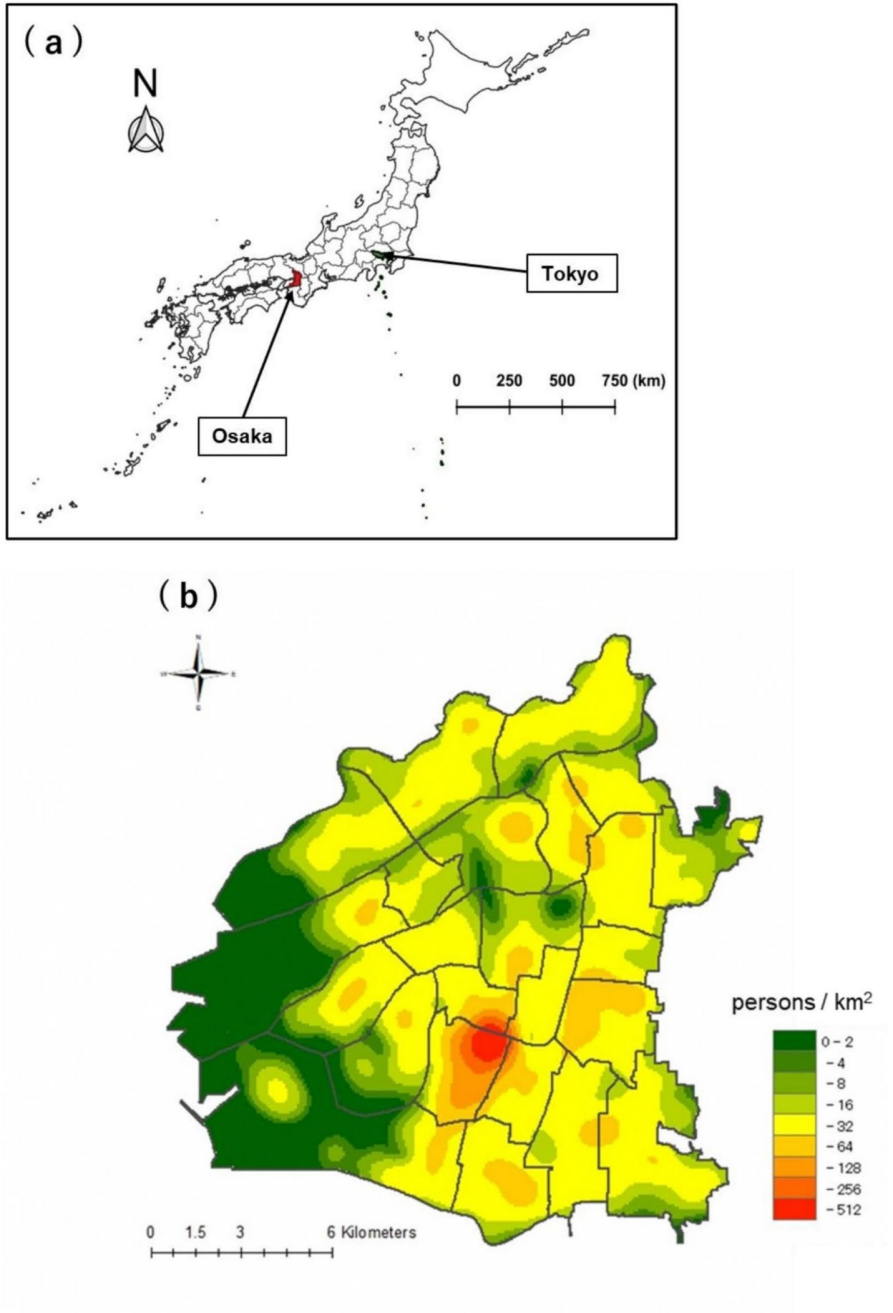
(**a**) Geographic location of Osaka City in Japan. The city is located about 600 km west of Tokyo and is the largest city economically in the western part of Japan. The map was created by QGIS 3.30 (QGIS Development Team, 2023, http://qgis.org). The administrative boundary data used in this study was obtained from the Humanitarian Data Exchange (HDX, https://data.humdata.org) and is provided under a CC BY-IGO license. (**b**) Kernel density estimation of tuberculosis patient addresses.

This study included a total of 4,852 individuals, representing over 97% of all patients with TB registered during the study period between 2012 and 2016 in Osaka City. The population was predominantly male, and individuals aged > 60 years accounted for approximately 70% of the sample, which was consistent with previously observed trends among patients with TB in Japan^10^. To illustrate the spatial distribution of patients with TB, a kernel density distribution map was generated (Fig. 1) based on the patient’s residence. The map revealed that sporadic TB cases occurred in all parts of the city. Additionally, high TB incidence was observed in a region (Airin) in the south-central part of the city. Airin, which has a high proportion of economically underprivileged residents, including residentially unstable day laborers and welfare recipients, has been recognized as a hotspot for TB cases^14^.

### Social area classification

**Fig. 2:**
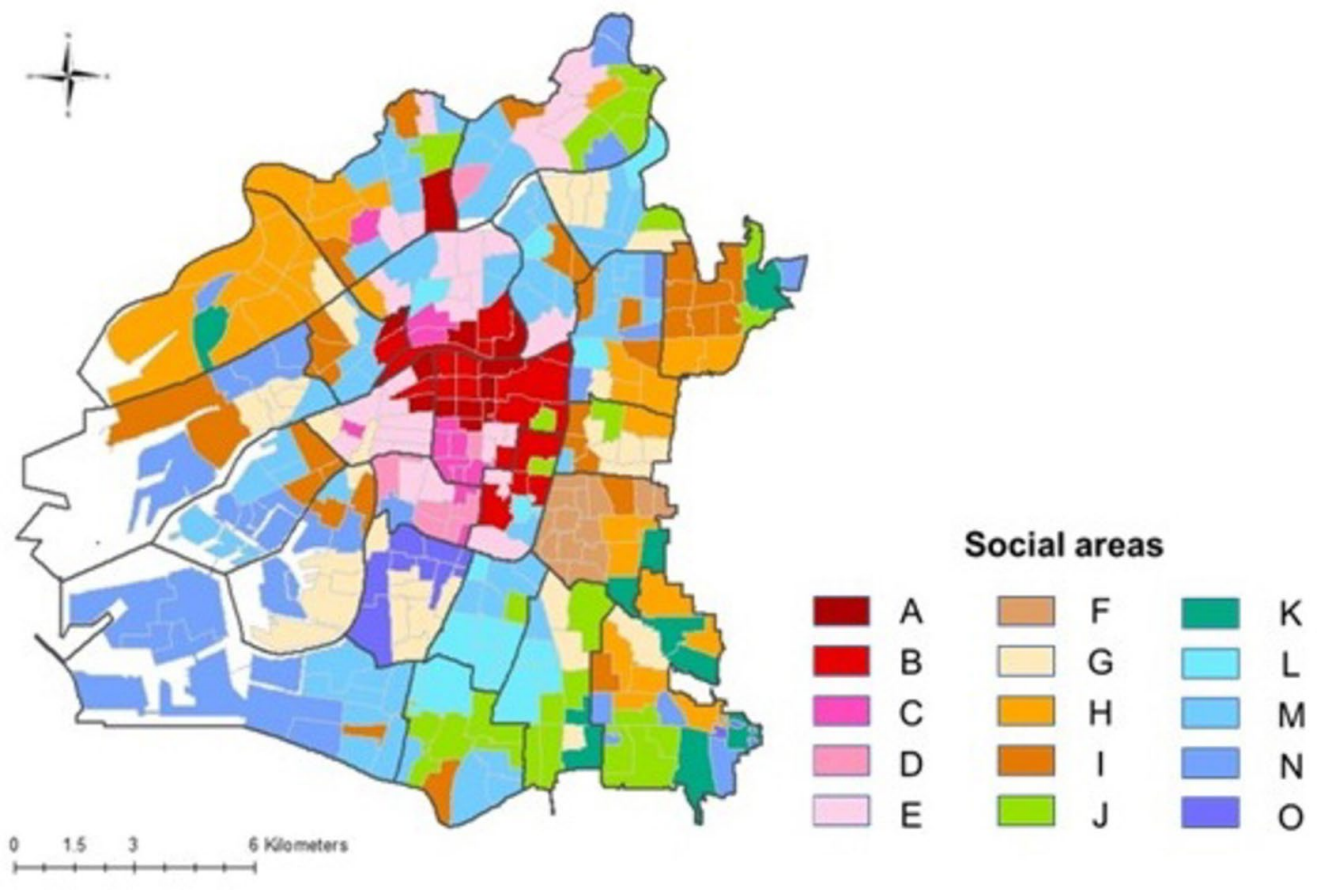
Geographical distribution of 15 social areas formed by clustering 332 town councils in Osaka City using factor analysis. Bold black lines indicate the 24 current administrative wards of the city.

To investigate the socioeconomic residential structure of the city, we conducted a factor analysis of the 42 indicators of town councils (Supplementary Table 1), which yielded six common factors with a cumulative contribution rate of 68.9%. The six factors were interpreted based on large absolute values of the factor loadings (Table 1). Using Ward’s hierarchical clustering with these factor scores, the 332 town councils were classified into 15 social areas (Fig. 2). A dendrogram of the clustering is shown in Supplementary Fig. 2.

**Table 1:** Estimated properties of the common factors of 332 town councils of Osaka City.

Factor 1	Related to higher education/employment in the tertiary sector / technical or managerial positions / younger individuals
Factor 2	Related to employment in the secondary sector / being married
Factor 3	Related to older adults / long-term residents
Factor 4	Related to lower education/service positions
Factor 5	Related to the residence of foreign people
Factor 6	Related to employment in the primary sector

The average factor scores of the town councils in each social area are summarized in Table 2. According to this clustering, all 24 administrative wards contained three to seven social areas, which means that they contained different socioeconomic residential neighborhoods as their constituents (Fig. 2).

**Table 2:** Scores of common factors and estimated characteristics of 15 social areas of Osaka City, Japan.

Social areas	Factor 1	Factor 2	Factor 3	Factor 4	Factor 5	Factor 6	Interpreted characteristics of residents to be noted*
A	0.61	− 1.39	− 1.68	− 1.66	− 0.18	− 0.20	young, not married, short residence, highly educated, technical/managerial positions
B	1.13	− 0.46	0.26	− 2.27	− 0.64	− 0.38	having real estate, highly educated, technical/managerial positions
C	0.82	− 2.1	0.79	1.04	1.73	0.35	not married, service positions
D	− 0.81	− 2.07	− 2.21	1.229	0.06	− 0.30	young, not married, short residence, service positions, foreigners
E	0.66	− 0.65	− 1.08	0.26	− 0.03	− 0.15	young, service positions
F	− 0.58	0.19	0.945	0.23	2.10	− 0.28	foreigner, older adults, having real estate, long residence, lower education
G	− 0.42	0.22	0.57	0.26	0.52	− 0.37	older adults, long residence, the secondary sector
H	− 0.73	1.11	-0.54	-0.62	0.77	− 0.31	married, the secondary sector
I	− 0.03	0.67	-0.69	0.27	0.11	− 0.59	the secondary sector
J	0.11	0.22	-0.04	0.22	-0.28	1.56	the secondary sector
K	− 0.74	0.73	− 0.29	− 0.63	0.56	3.58	married, mixture with the primary and secondary sectors
L	1.26	0.26	1.63	− 0.62	− 0.25	0.05	older adults, married, having real estate, highly educated, technical/managerial positions
M	0.46	0.095	0.20	0.49	− 0.26	− 0.17	Occupational diversity, White-collar enclave
N	− 0.44	0.47	0.47	0.67	− 1.77	− 0.44	married, long residence, lower education, the secondary sector
O	− 2.79	− 1.54	1.26	− 0.71	− 0.77	− 0.06	older adults, not married, short residence, low educated
* Estimated by the factor scores of each area (Table 1) and then verified according to the census data of residence.							

### Regional differences in TB incidence by social area

Standardized TB incidence ratios were significantly higher in two areas (Areas D and O) (Table 3). Area O includes “Airin,” an area with extremely high TB cases. The residents in this area were older, single, short-term residents, and less educated (Table 2). Area D is located in and around “Minami,” a downtown area of Osaka City, adjacent to Area O. Based on the factor scores (Table 2), this area was interpreted as having a large number of young adults, singles, short-term residents, service industry workers, and foreign-born residents (Table 2).

**Table 3:** Standardized TB incidence ratio of respective social areas during 2012–2016.

Social area	Number of the total population*	Expected number of patients with TB per year**	Number of patients with TB per year	Standardized TB incidence ratio (95%CI)
A	45,895	13.4	9.8	0.73 (0.37–1.4)
B	91,042	29.1	19.8	0.68 (0.43–1.1)
C	26,860	10.1	14.6	1.4 (0.83–2.5)
D	50,602	17.0	31.2	1.8 (1.3–2.6)
E	234,951	74.1	62.8	0.85 (0.66–1.1)
F	85,051	35.8	35.2	0.98 (0.69–1.4)
G	246,945	97.5	115.6	1.2 (0.99–1.4)
H	280,143	95.6	78.0	0.82 (0.65–1.0)
I	259,842	85.5	75.4	0.88 (0.7–1.1)
J	252,215	90.7	75.6	0.83 (0.66–1.1)
K	81,115	27.2	25.8	0.95 (0.63–1.4)
L	160,890	61.0	43.4	0.71 (0.52–0.96)
M	536,855	194.9	155.0	0.8 (0.68–0.94)
N	223,448	84.9	81.8	0.96 (0.77–1.2)
O	65,506	31.7	146.4	4.6 (3.9–5.5)
Total	2,646,794	-	998.0	1

* The population size of each social area was calculated by using the census data of 2010, excluding those of unknown age.

TB: tuberculosis, CI: confidence interval.

Conversely, two areas (Areas L and M) had a significantly lower ratio (Table 3): Area L had a large population of affluent older adult long-term residents, while Area M had many white-collar commuters with different socioeconomic statuses and industrial composition residents (Table 2).

### Characteristics of patients in social areas by TB incidence

With the goal of exploring the social area factors causing higher standardized TB incidence, we examined the regional characteristics thoroughly in the two regions with the highest ratios (Areas D and O). A multinomial logistic regression analysis was performed to examine the characteristics of patients with TB living in these areas. The characteristics of patients with TB were as follows. Regarding age, patients in Area D were more likely to be < 60 years, whereas those in Area O were more likely to be 40–79 years. Significant differences were observed in the number of foreign-born patients, with more patients in Area D than in Area O. Considering the history of TB diagnosis, patients residing in Area D were often diagnosed while undergoing treatment for other diseases. Patients living in Area O were more likely to be registered as recurrent cases; moreover, fewer patients had a delayed diagnosis of > 3 months (Table 4).

**Table 4:** Multinomial logistic regression analysis for examining the characteristics of patients with TB living in social areas with high standardized TB incidence ratios.

	Number of patients with TB			D vs. The other social areas		O vs. The other social areas	
	D	O	The other social areas	AOR (95% CI)	p value	AOR (95% CI)	p value
	(*n* = 156)	(*n* = 732)	(*n* = 3,964)				
Sex							
Male	119 (76.3%)	669 (91.4%)	2,575 (65.0%)	1.8 (1.3–2.7)	*p* = 0.0018	4.7 (3.5–6.1)	*p* < 0.001
Female	37 (23.7%)	63 (8.6%)	1,389 (35.0%)	Ref		ref	
Age group							
< 40	37 (23.7%)	29 (4.0%)	533 (13.4%)	2.8 (1.6–4.9)	*p* < 0.001	0.66 (0.42–1.0)	*p* = 0.068
40–59	36 (23.1%)	153 (20.9%)	749 (18.9%)	2.0 (1.2–3.3)	*p* = 0.0093	1.9 (1.5–2.6)	*p* < 0.001
60–79	53 (34.0%)	448 (61.2%)	1,576 (39.8%)	1.2 (0.77–1.9)	*p* = 0.39	2.6 (2.0–3.3)	*p* < 0.001
≥ 80	30 (19.2%)	102 (13.9%)	1,106 (27.9%)	Ref		Ref	
National origin							
Domestic	128 (82.1%)	689 (94.1%)	3,389 (85.5%)	Ref		Ref	
Foreign	14 (9.0%)	4 (0.55%)	152 (3.8%)	2.0 (1.0–3.7)	*p* = 0.038	0.25 (0.090–0.70)	*p* = 0.0085
Unknown	14 (9.0%)	39 (5.3%)	423 (10.7%)	0.92 (0.52–1.6)	*p* = 0.78	0.38 (0.26–0.56)	*p* < 0.001
Respiratory symptom							
Positive	82 (52.6%)	390 (53.3%)	2,078 (52.4%)	1.0 (0.74–1.5)	*p* = 0.85	0.99 (0.83–1.2)	*p* = 0.88
Negative	73 (46.8%)	310 (42.3%)	1,817 (45.8%)	Ref		Ref	
Unknown	1 (0.6%)	32 (4.4%)	69 (1.7%)	0.50 (0.062–4.1)	*p* = 0.52	2.3 (1.3–4.0)	*p* = 0.0061
History of medical treatment							
Initial treatment	145 (92.9%)	605 (82.7%)	3,546 (89.5%)	Ref		Ref	
Recurrence	9 (5.8%)	105 (14.3%)	336 (8.5%)	0.65 (0.33–1.3)	*p* = 0.21	1.5 (1.2–1.9)	*p* = 0.0022
Unknown	2 (1.3%)	22 (3.0%)	82 (2.1%)	0.91 (0.21–4.0)	*p* = 0.90	0.94 (0.51–1.7)	*p* = 0.85
History of TB diagnosis							
Medical consultation	86 (55.1%)	378 (51.6%)	2,336 (58.9%)	Ref		Ref	
Physical examination	23 (14.7%)	88 (12.0%)	536 (13.5%)	0.76 (0.45–1.3)	*p* = 0.29	1.2 (0.87–1.5)	*p* = 0.33
Under treatment of other diseases	45 (28.8%)	173 (23.6%)	964 (24.3%)	1.5 (1.1–2.3)	*p* = 0.027	1.1 (0.88–1.3)	*p* = 0.46
Unknown	2 (1.3%)	93 (12.7%)	128 (3.2%)	0.39 (0.089–1.7)	*p* = 0.21	4.3 (3.0–6.0)	*p* < 0.001
Delayed diagnosis of TB							
More than 3 months	29 (18.6%)	124 (16.9%)	944 (23.8%)	0.71 (0.47–1.1)	*p* = 0.11	0.64 (0.51–0.79)	*p* < 0.001
Less than 3 months	122 (78.2%)	595 (81.3%)	2931 (73.9%)	Ref		Ref	
Unknown	5 (3.2%)	13 (1.8%)	89 (2.2%)	1.7 (0.67–4.5)	*p* = 0.26	0.27 (0.13–0.58)	*p* < 0.001

TB: tuberculosis, AOR: adjusted odds ratio, CI: confidence interval, Delayed diagnosis of TB: Delay in diagnosis from symptom onset.

All variables have a $\text{GVIF}^{(1/(2Df))}$ value below 3, indicating that multicollinearity is not a concern in this model.

## Discussion

This comparative study of TB incidence among social areas classified using geographic segmentation revealed the potential for geodemographic analysis to represent TB incidence patterns across cities more accurately. In this study, Osaka City was found to have significantly higher TB incidence rates limited to two social areas that vary in nature. Area O had the highest TB incidence rate (146.4 per 65,506 population), higher than that of the highest-level administrative ward, Nishinari (180.6 per 100,000 population, 2012–2016 average). These findings indicate that geographic classifications can identify TB hotspots more accurately compared to traditional administrative boundaries.

Area O, an identified TB hotspot, encompasses Airin—a historic urban center known for its concentration of day laborers^15^. Unlike regions where immigrants and people living with human immunodeficiency virus (HIV) are typically considered the primary TB risk groups^16^, Airin has a unique profile. Here, foreign-born patients and people living with HIV accounted for only 0.55% and 0.41% (data not shown) of the TB cases, respectively, which starkly contrasts with international patterns. Paradoxically, less delay in TB detection was observed in Airin, which could be attributed to the enhanced screening program in this area. Despite the introduction of an effective detection system, TB incidence rates remain at an alarmingly high level. This persistent transmission is likely due to district-specific environmental factors. Specifically, the presence of communal spaces and homeless shelters, the spread of localized infections confirmed by recent genetic studies of patient isolates^17^, and the higher prevalence of latent TB infection compared to the general Japanese population^18^ all contribute to the sustained transmission. Thus, the distinctive TB situation in area O, particularly within the Airin area, underscores the importance of targeted interventions to disrupt local transmission chains. This unique study offers valuable insights into urban public health strategies and highlights the need for context-specific approaches for TB control.

Delays in TB diagnosis may be influenced by socioeconomic factors, including patient recall bias and disparities in healthcare access^19,20^. This potential bias necessitates a cautious interpretation of the disparities observed in area O (Table 4). While Japan’s urban areas have historically exhibited less pronounced socioeconomic gaps compared to those in Western countries, recent trends indicate a significant widening of these disparities. For instance, Tokyo is experiencing increasing residential segregation based on occupation^21^. This shift is particularly relevant to TB, given its strong correlation with socioeconomic status^22,23^. To address these evolving dynamics, future TB research should incorporate more nuanced, region-specific analyses. Researchers should remain vigilant of confounding factors arising from demographic variations linked to widening regional disparities. These considerations are crucial for developing effective targeted TB control strategies that account for the complex interplay between socioeconomic factors and disease prevalence.

Considering the demographic changes, a detailed geographic analysis of TB incidence could provide valuable insights. Area D stands out for its large number of young foreign-born and short-term residents. Notably, a significant proportion of TB cases in this region included young foreigners (Table 4). Although surveillance data from Tokyo currently show limited cases of TB infection originating from foreign-born individuals, highlighting its unlikely role as a major risk factor in Japan^24^, the recent surge in foreign-born arrivals cannot be overlooked. The increased proportion of foreign-born patients with TB has highlighted the importance of foreign-born patients with TB in Japan’s TB control strategies. By examining the spatial distribution of patients in different population groups, we can better understand the unique challenges faced by regions such as Area D and adjust relevant strategies accordingly.

Combining a geodemographic analysis of TB incidence with a genotypic analysis of clinical strains of *M. tuberculosis* may provide valuable insights into the transmission dynamics of TB and help adjust strategies accordingly. As a limitation of the study, it is important to note that areas D and O, which showed significantly higher TB incidences, were geographically adjacent to each other. This suggests the possibility of large-scale transmission in this area, warranting further investigation. In addition, endogenous reactivation and recent transmission cases could not be differentiated based on patient data. Such a distinction could help identify geographic areas prone to TB outbreaks and enable the development of targeted and effective interventions. Because this is a cross-sectional study, only basic demographic data can be presented, and a causal relationship cannot be definitively established. Although an association was found between local residents’ demographic characteristics and TB cases, regional differences are likely to fluctuate over time. Therefore, continuous reanalysis and comparative studies using the most recent data are essential to examine the variation in TB incidence more comprehensively.

## Conclusions

Our geodemographic analysis of patients with TB in Osaka City revealed distinct socioeconomic patterns in neighboring areas with high TB incidences. This approach, which considers the social characteristics of each region, offers valuable insights into the development of targeted and effective TB control strategies. These analyses are meaningful in urban environments with localized social disparities, enabling tailored interventions to reduce TB incidence while accounting for each city’s unique social characteristics.

## Methods

### Geodemographic clustering of social areas

In this study, data was obtained from the public resources of the Osaka City Planning and Coordination Bureau^25^. Based on these tabulation data of Osaka City from the 2010 National Census, social area clustering was established. Osaka City has 332 town councils. The Z-score normalization for 42 indicators reflected the familial, socioeconomic, and ethnic statuses of the residents of each town council (Supplementary Table 1). These indicators of the abovementioned three statuses have traditionally been employed in various studies on social area analysis and factorial ecology in urban sociology and geography^26,27^. We applied factor analysis to the 42 indicators using the maximum likelihood method and varimax rotation to extract common factors. Geodemographic clustering of the 332 town councils into social areas was performed using Ward’s method and the scores of common factors.

### Geocoding of the residence of patients with TB

Data on TB patients was obtained from the Osaka City Public Health Office. The number of patients with TB who lived in each social area and were registered as new cases in Osaka City between 2012 and 2016 was counted based on their residential address. The town councils of 4,990 patients with TB were identified from their residential addresses, but those of 138 patients could not be detected. Consequently, data from 4,852 individuals were used in this study.

### Standardization of TB incidence ratio in social areas

The standardized annual TB incidence ratio for each social area (*S*_*i*_, where *i* denotes the respective social area) during the study period was calculated by dividing the number of registered TB cases in each area (*P*_*i*_) by the expected number of TB cases per year (*E*_*i*_).

*S*_*i*_ = *P*_*i*_/*E*_*i*_.

Here, *E*_*i*_ was calculated based on the ratio of the population of each area (*N*_*i*_) to the total population of Osaka City (*N*_*t*_) from the total number of registered patients with TB (4,852 persons) in the entire city. It was calculated based on the age distribution of registered patients with TB (Supplementary Table 2) to consider the age structure of each area. Hence, for a given age group *j* (in increments of 10 years), if the population of Osaka City as a whole is *N*_*tj*_, the population of each area is *N*_*ij*_, and the number of patients with TB in Osaka City as a whole is *P*_*tj*_, then *E*_*i*_ is the sum of all age groups as follows:

$$\:{E}_{i}\:=\:{\sum\:}_{j}^{}{N}_{ij}\left({P}_{tj}/{N}_{tj}\right)$$

The 95% confidence interval for *Si* was calculated based on a Poisson distribution^28^.

### Characteristics of patients with TB in social areas with significantly higher standardized TB incidence ratios

Multinomial logistic regression was used to examine the characteristics of patients with TB in social areas where the standardized TB incidence ratio was significantly higher. Two social areas with high TB incidence ratios and other social areas were used as objective variables. The explanatory variables selected for the study included sex, age group, national origin, respiratory symptoms, history of medical treatment, history of TB diagnosis, and delay in the diagnosis from symptom onset (Table 4). The adjusted odds ratio, along with the 95% confidence interval and p-value, was calculated after adjusting for all explanatory variables.

### Data analysis

R ver. 3.4.3 (R Foundation for Statistical Computing, Vienna, Austria) was used for the statistical analysis. ArcGIS ver. 10.5 (ESRI, Redlands, CA) was used for mapping, unless otherwise noted in the figure legend.

### Ethical considerations

This study was a retrospective observational study. Two types of data were used: one, small area aggregated results from the national census obtained from the Osaka City Planning and Coordination Bureau; two, tuberculosis patient data collected by the Osaka City Health Center through active epidemiological investigation based on the Infectious Diseases Control Law.

This study was conducted in accordance with the “Ethical Guidelines for Life Science and Medical Research Involving Human Subjects” established by the Ministry of Health, Labour, and Welfare of Japan (effective March 23, 2021). Ethical approval was obtained from the Ethical Review Committee of the first author’s institution (approval number: 1812-01-4, approval date: April 1, 2023).

As this study was a retrospective observational study using existing data, the requirement for obtaining individual informed consent was waived. However, in accordance with the aforementioned ethical guidelines, an overview of the research and opt-out procedures was posted on the institution’s website and made publicly available for a specified period. During this period, no requests to opt out of the study were received.

All information handled in this study was anonymized and did not contain personally identifiable information. Data handling strictly adhered to the policies of the Ethics Committee, and the analysis was conducted in a secure environment. Address data were converted to coordinate values in a stand-alone environment to avoid the risk of online data breach, using the basic geocoding function of ArcGIS Pro 2.4 (ESRI, Redlands, CA) with the Japan Data Contents Starter Pack (2018) (ESRI Japan, Tokyo, Japan).

## Electronic supplementary material

Below is the link to the electronic supplementary material.

Supplementary Material 1

## Data Availability

The tabulation data of Osaka City from the 2010 National Census and shapefiles of the 332 town councils in Osaka City are not publicly available but can be obtained from the Urban Planning Bureau of Osaka City (https://www.city.osaka.lg.jp/toshikeikaku/) upon reasonable request. The patient information obtained through the Infectious Disease Surveillance under the Infectious Diseases Control Law is not publicly available due to the sensitive nature of the data and restrictions on access. The data may not be available for public use due to legal and privacy restrictions, and is generally not accessible.
