# Supplementary material for: Geodemographic analysis of socioeconomic area disparities in tuberculosis incidence in Osaka City, Japan
Source: Sci Rep. 2025 May 7;15:15968. doi: 10.1038/s41598-025-99711-4 (PMC12059140; doi:10.1038/s41598-025-99711-4)
Supplement: Supplementary file 1 — Supplementary Material 1 [file 41598_2025_99711_MOESM1_ESM.docx]

**Geodemographic analysis of socioeconomic area disparities in tuberculosis incidence in Osaka City, Japan**

Kaori Yamamoto^a^, Shouhei Takeuchi^b^, Tomoki Nakaya^c,d^, Naoya Fujiwara^e,f^, Junji Seto^g^, Jun Komukai^h^, Yuko Tsuda^h^, Hideki Yoshida^i^, and Takayuki Wada^j,k^

^a^ Division of Microbiology, Osaka Institute of Public Health, Osaka, Japan

^b^ Faculty of Nursing and Nutrition, University of Nagasaki, Nagayo, Nishisonogi, Japan

^c^ Graduate School of Environmental Studies, Tohoku University, Sendai, Japan

^d^ Department of Earth Science, Graduate School of Science, Tohoku University, Sendai, Japan

^e^ Graduate School of Information Sciences, Tohoku University, Sendai, Japan

^f^ PRESTO, Japan Science and Technology Agency, Kawaguchi, Japan

^g^ Department of Microbiology, Yamagata Prefectural Institute of Public Health, Yamagata, Japan

^h^ Department of Infectious Disease Control, Osaka City Public Health Office, Osaka, Japan

^i^ Osaka City Health Bureau, Osaka, Japan

^j^ Graduate School of Human Life and Ecology, Osaka Metropolitan University, Osaka, Japan

^k^ Osaka International Research Center for Infectious Diseases, Osaka Metropolitan University, Osaka, Japan

| **Supplementary Table 1** The 42 indicators for factor analysis to characterize the socioeconomic situation of 332 areas in Osaka City, Japan | | | | | | | | | |
| --- | --- | --- | --- | --- | --- | --- | --- | --- | --- |
| Indicator | |  | Common factor | | | | | | |
| Category | Property |  | Factor 1 | Factor 2 | Factor 3 | Factor 4 | Factor 5 | Factor 6 | Commonality |
| Population | Male |  | -0.562 | -0.413 | 0.029 | -0.099 | -0.028 | -0.034 | 0.499 |
|  | Aged 0–14 y |  | 0.052 | 0.898 | -0.128 | 0.053 | -0.064 | 0.148 | 0.854 |
|  | Aged 15–29 y |  | 0.393 | -0.418 | -0.660 | -0.013 | 0.190 | -0.045 | 0.803 |
|  | Aged 30–44 y |  | 0.519 | -0.036 | -0.681 | -0.365 | -0.042 | -0.023 | 0.870 |
|  | Aged 45–59 y |  | 0.087 | -0.141 | 0.446 | -0.267 | -0.086 | -0.136 | 0.324 |
|  | Aged 60–74 y |  | -0.660 | -0.152 | 0.628 | 0.245 | -0.101 | -0.003 | 0.923 |
|  | Aged ≥ 75 y |  | -0.325 | -0.092 | 0.717 | 0.258 | 0.099 | 0.011 | 0.705 |
|  | Foreign residents |  | -0.238 | -0.116 | 0.006 | 0.021 | 0.476 | -0.064 | 0.301 |
|  | Married by age* |  | 0.229 | 0.940 | 0.133 | 0.004 | -0.111 | 0.112 | 0.979 |
|  | Population Density |  | -0.007 | -0.017 | 0.010 | 0.175 | -0.058 | -0.046 | 0.037 |
| Residence | Owned houses |  | 0.311 | 0.551 | 0.329 | -0.169 | 0.368 | -0.062 | 0.676 |
|  | Rented houses owned by governmental or quasi-governmental entities |  | -0.265 | 0.202 | 0.134 | 0.215 | -0.631 | 0.040 | 0.575 |
|  | Rented house owned by private company |  | -0.015 | -0.656 | -0.398 | -0.043 | 0.361 | 0.021 | 0.722 |
|  | Housing for company employees and civil servants |  | 0.364 | -0.182 | -0.184 | -0.431 | -0.033 | -0.113 | 0.399 |
|  | Rented rooms |  | -0.130 | -0.098 | 0.139 | 0.064 | 0.514 | -0.092 | 0.323 |
|  | Resided in the same place since birth |  | 0.067 | 0.371 | 0.131 | 0.138 | 0.707 | 0.067 | 0.683 |
|  | Resided in current place for less than a year |  | 0.151 | -0.602 | -0.386 | -0.320 | -0.191 | -0.030 | 0.674 |
|  | Resided in current place for 20 years or more |  | -0.236 | 0.104 | 0.576 | 0.433 | 0.308 | -0.041 | 0.682 |
| Working* | Workers in primary industries |  | -0.142 | 0.185 | -0.011 | -0.030 | -0.071 | 0.907 | 0.883 |
|  | Workers in secondary industries |  | -0.614 | 0.717 | 0.012 | -0.055 | 0.155 | 0.104 | 0.929 |
|  | Workers in tertiary industries |  | 0.862 | -0.272 | 0.061 | 0.121 | -0.246 | -0.100 | 0.906 |
|  | Administrative and managerial workers |  | 0.565 | -0.406 | 0.040 | -0.487 | 0.145 | -0.047 | 0.746 |
|  | Professional and engineering workers |  | 0.753 | -0.118 | -0.133 | -0.462 | -0.078 | -0.066 | 0.823 |
|  | Clerical workers |  | 0.712 | 0.329 | -0.130 | -0.238 | -0.240 | -0.108 | 0.758 |
|  | Sales workers |  | 0.723 | -0.243 | -0.141 | -0.002 | 0.260 | 0.014 | 0.669 |
|  | Service workers |  | -0.058 | -0.574 | 0.177 | 0.511 | 0.260 | 0.020 | 0.693 |
|  | Security workers |  | -0.114 | 0.060 | 0.154 | 0.125 | -0.322 | -0.025 | 0.160 |
|  | Agriculture, forestry, and fishery workers |  | -0.179 | 0.250 | -0.060 | 0.014 | -0.093 | 0.910 | 0.935 |
|  | Manufacturing process workers |  | -0.595 | 0.660 | 0.047 | 0.136 | 0.218 | 0.101 | 0.868 |
|  | Transport and machine operation workers |  | -0.496 | 0.524 | 0.098 | 0.370 | -0.334 | 0.019 | 0.779 |
|  | Construction and mining workers |  | -0.686 | 0.206 | 0.093 | 0.031 | -0.119 | 0.095 | 0.546 |
|  | Carrying, cleaning, packaging, and related workers |  | -0.621 | 0.270 | 0.209 | 0.445 | -0.400 | -0.007 | 0.860 |
| Educational background** | Elementary school, junior high school |  | -0.870 | 0.093 | 0.249 | 0.309 | -0.145 | 0.062 | 0.948 |
|  | Senior high school |  | -0.515 | 0.396 | 0.197 | 0.644 | 0.173 | 0.063 | 0.909 |
|  | Junior college or higher professional school |  | 0.787 | 0.020 | -0.439 | -0.243 | 0.052 | -0.032 | 0.875 |
|  | College, university, or graduate course |  | 0.743 | -0.319 | -0.170 | -0.553 | -0.004 | -0.077 | 0.994 |
| Means of Transport*** | Only on foot |  | 0.430 | -0.482 | -0.145 | -0.503 | 0.146 | -0.138 | 0.732 |
|  | Train, subway, tram, public or private |  | 0.616 | 0.280 | -0.100 | -0.145 | -0.220 | -0.092 | 0.546 |
|  | Bus |  | -0.112 | 0.302 | 0.040 | 0.092 | -0.154 | -0.201 | 0.178 |
|  | Private car |  | -0.151 | 0.847 | 0.054 | 0.030 | -0.197 | 0.191 | 0.819 |
|  | Motorcycle |  | -0.348 | 0.741 | -0.127 | 0.142 | -0.046 | 0.207 | 0.751 |
|  | Bicycle |  | -0.119 | 0.719 | -0.243 | 0.151 | 0.031 | 0.098 | 0.624 |
| Eigenvalue (after varimax rotation) | |  | 9.286 | 8.073 | 3.492 | 3.31 | 2.817 | 1.981 |  |
| Contribution ratio (%) | |  | 22.1 | 19.2 | 8.3 | 7.9 | 6.7 | 4.7 |  |
| Cumulative contribution ratio (%) | |  | 22.1 | 41.3 | 49.6 | 57.5 | 64.2 | 68.9 |  |
| * Survey participants are 15 years old and over.  ** Survey participants are 15 years of age and over and have graduated.  *** Means of transport refer to those which are usually used to go to work or to attend school. Survey participants are 15 years old and over. | | | | | | | | | |

| **Supplementary Table 2** Attributes of all patients with TB included in this study | | | | |
| --- | --- | --- | --- | --- |
| Attributes | | *n* (%) |  |  |
| ***Total*** | | 4,852 |  |  |
| ***Sex*** | |  |  |  |
|  | Male | 3,363 (69.3) |  |  |
|  | Female | 1,489 (30.7) |  |  |
| ***Age group*** | |  |  |  |
|  | < 40 | 599 (12.4) |  |  |
|  | 40–59 | 938 (19.3) |  |  |
|  | 60–79 | 2,077 (42.8) |  |  |
|  | ≥ 80 | 1,238 (25.5) |  |  |
| ***National origin*** | | | | |
|  | Domestic | 4,206 (86.7) |  |  |
|  | Foreign | 170 (3.5) |  |  |
|  | Unknown | 476 (9.8) |  |  |
| ***Pulmonary/extrapulmonary*** | |  |  |  |
|  | Pulmonary | 4,213 (86.8) |  |  |
|  | Extrapulmonary | 639 (13.2) |  |  |
| ***Respiratory symptom*** | |  |  |  |
|  | Positive | 2,550 (52.6) |  |  |
|  | Negative | 2,200 (45.3) |  |  |
|  | Unknown | 102 (2.1) |  |  |
| ***History of medical treatment*** | |  |  |  |
|  | Initial treatment | 4,296 (88.5) |  |  |
|  | Recurrence | 450 (9.3) |  |  |
|  | Unknown | 106 (2.2) |  |  |
| ***History of TB diagnosis*** | |  |  |  |
|  | Medical consultation | 2,800 (57.7) |  |  |
|  | Physical examination | 647 (13.3) |  |  |
|  | Under treatment of other diseases | 1,182 (24.4) |  |  |
|  | Unknown | 223 (4.6) |  |  |
| ***Delayed diagnosis of TB*** | |  |  |  |
|  | More than 3 months | 1097 (22.6) |  |  |
|  | Less than 3 months | 3,648 (75.2) |  |  |
|  | Unknown | 107 (2.2) |  |  |
| TB: tuberculosis | | |  |  |

**
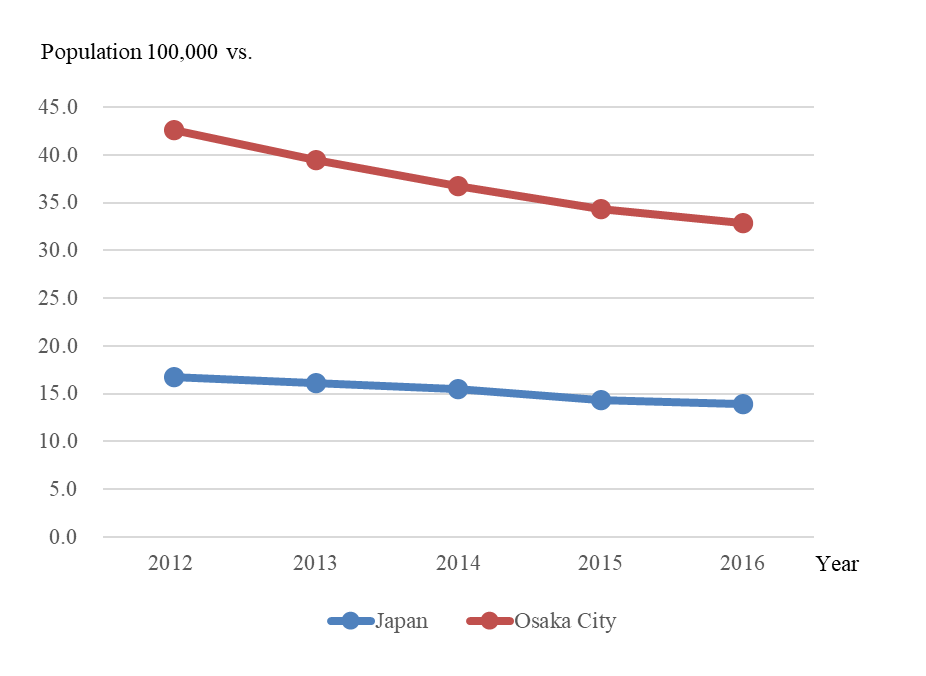
Supplementary Fig. 1**

The TB incidence rate (per 100,000 population) decreased from 16.7 in 2012 to 13.9 in 2016 in Japan. The TB incidence rate in Osaka City decreased from 42.7 in 2016 to 32.8 in 2016. The TB incidence rate in Osaka City is approximately twice as high as that in Japan.

**
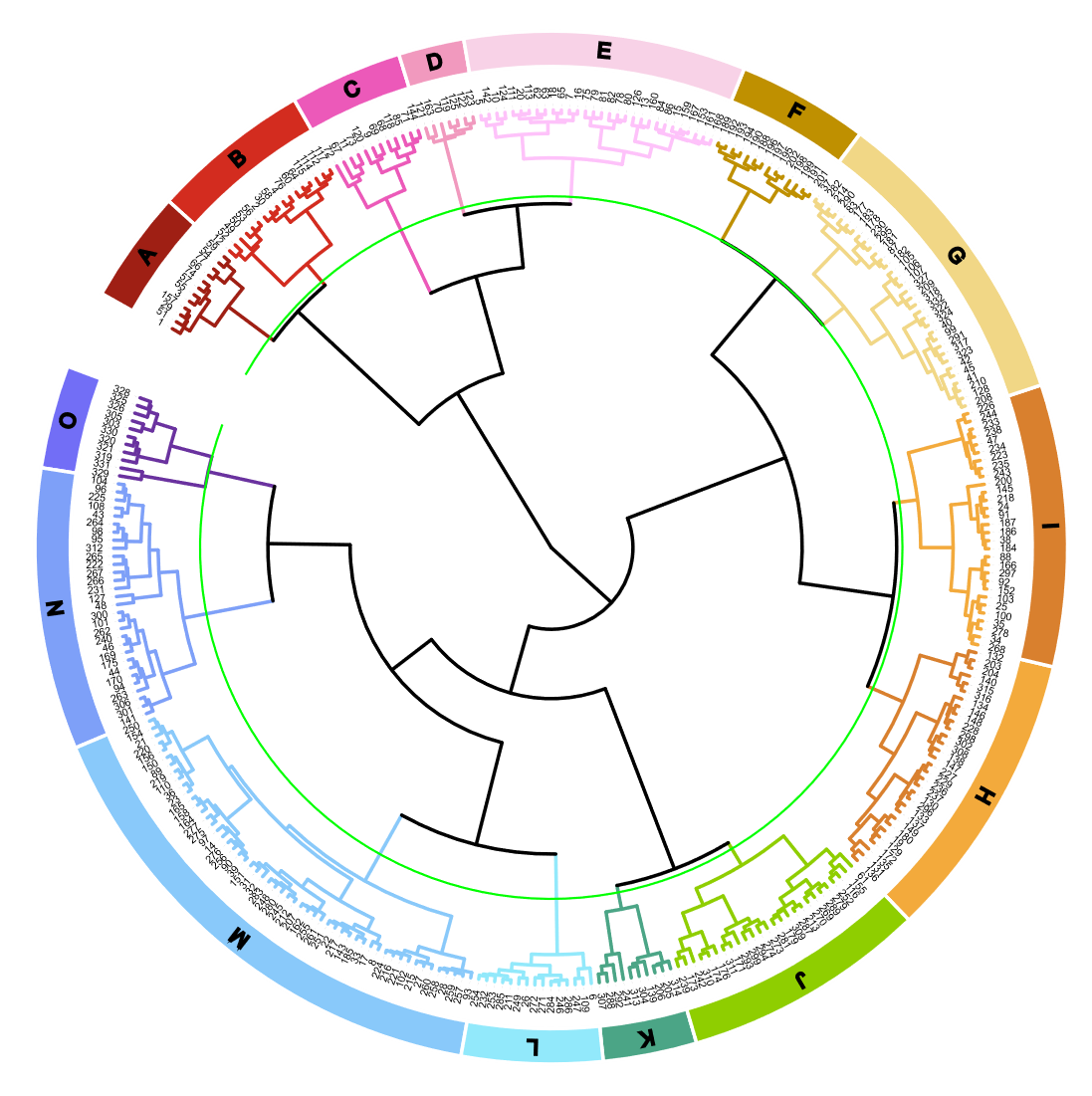
Supplementary Fig. 2**

A dendrogram of the 332 town councils of Osaka City, based on their respective factor scores calculated by factor analysis on census data of residents. The label number indicates the town councils. Each color of the branches and the belt around the perimeter of the tree corresponds to the map coloring shown in Fig. 2. The scale corresponds to the height of the dendrogram created using the hclust package of R ver. 3.4.3. The horizontal light green line represents the division threshold for the 15 clusters (social areas). The tree was visualized using iTOL ver. 6.7.6 [(Letunic and Bork 2021)](https://paperpile.com/c/Fskn96/QpSX).
